# Subjective cognitive decline predicts longitudinal neuropsychological test performance in an unsupervised online setting in the Brain Health Registry

**DOI:** 10.1186/s13195-024-01641-2

**Published:** 2025-01-07

**Authors:** Jae Myeong Kang, Manchumad Manjavong, Chengshi Jin, Adam Diaz, Miriam T. Ashford, Joseph Eichenbaum, Emily Thorp, Elizabeth Wragg, Kenton H. Zavitz, Francesca Cormack, Anna Aaronson, R. Scott Mackin, Rachana Tank, Bernard Landavazo, Erika Cavallone, Diana Truran, Sarah Tomaszewski Farias, Michael W. Weiner, Rachel L. Nosheny

**Affiliations:** 1 Department of Psychiatry and Behavioral Sciences, University of California San Francisco, San Francisco, CA USA; 2 VA Advanced Imaging Research Center, San Francisco Veteran’s Administration Medical Center, San Francisco, CA USA; 3 Department of Psychiatry, Gil Medical Center, Gachon University College of Medicine, Incheon, Republic of Korea; 4 Division of Geriatric Medicine, Department of Internal Medicine, Faculty of Medicine, Khon Kaen University, Khon Kaen, Thailand; 5 Department of Epidemiology and Biostatistics, University of California San Francisco, San Francisco, CA USA; 6 Northern California Institute for Research and Education (NCIRE), San Francisco, CA USA; 7 Department of Radiology and Biomedical Imaging, University of California San Francisco, San Francisco, CA USA; 8 Cambridge Cognition, Cambridge, UK; 9 Cambridge Cognition, Cambridge, MA USA; 10 Department of Psychiatry, University of Cambridge, Cambridge, UK; 11 Dementia Research Centre, UCL Institute of Neurology, University College London, London, WC1E 6BT UK; 12 Departments of Neurology, University of California Davis, Sacramento, CA USA; 13 Department of Neurology, University of California San Francisco, San Francisco, CA USA; 14 Department of Medicine, University of California San Francisco, San Francisco, CA USA

**Keywords:** Subjective cognitive decline, Everyday cognition scale, Paired associates learning, Brain health registry, Digital cognitive assessment

## Abstract

**Backgrounds:**

Digital, online assessments are efficient means to detect early cognitive decline, but few studies have investigated the relationship between remotely collected subjective cognitive change and cognitive decline. We hypothesized that the Everyday Cognition Scale (ECog), a subjective change measure, predicts longitudinal change in cognition in the Brain Health Registry (BHR), an online registry for neuroscience research.

**Methods:**

This study included BHR participants aged 55 + who completed both the baseline ECog and repeated administrations of the CANTAB^®^ Paired Associates Learning (PAL) visual learning and memory test. Both self-reported ECog (Self-ECog) and study partner-reported ECog (SP-ECog), and two PAL scores (first attempt memory score [FAMS] and total errors adjusted [TEA]) were assessed. We estimated associations between multiple ECog scoring outputs (ECog positive [same or above cut-off score], ECog consistent [report of consistent decline in any item], and total score) and longitudinal change in PAL. Additionally we assessed the ability of ECog to identify ‘decliners’, who exhibited the worst PAL progression slopes corresponding to the fifth percentile and below.

**Results:**

Participants (*n* = 16,683) had an average age of 69.07 ± 7.34, 72.04% were female, and had an average of 16.66 ± 2.26 years of education. They were followed for an average of 2.52 ± 1.63 visits over a period of 11.49 ± 11.53 months. Both Self-ECog positive (estimate = -0.01, *p* < 0.001, R²m = 0.56) and Self-ECog consistent (estimate=-0.01, *p* = 0.002, R²m = 0.56) were associated with longitudinal change in PAL FAMS after adjusting demographics and clinical confounders. Those who were Self-ECog total (Odds ratio [95% confidence interval] = 1.390 [1.121–1.708]) and SP-ECog consistent (2.417 [1.591–3.655]) had higher probability of being decliners based on PAL FAMS.

**Conclusion:**

In the BHR’s unsupervised online setting, baseline subjective change was feasible in predicting longitudinal decline in neuropsychological tests. Online, self-administered measures of subjective cognitive change might have a potential to predict objective subjective change and identify individuals with cognitive impairments.

**Supplementary Information:**

The online version contains supplementary material available at 10.1186/s13195-024-01641-2.

## Background

Alzheimer’s disease (AD) is characterized as amyloid plaques, tau tangles, and neurodegeneration, leading to cognitive decline and dementia [1]. AD can be diagnosed with biomarkers including amyloid positron emission tomography (PET) scans [2, 3], tau PET scans [4, 5], analysis of cerebrospinal fluid [6], and plasma testing [6, 7]. During the last year, Eisai’s monoclonal antibody called Leqembi has been approved by FDA and CMS in the USA [8]. The development of disease-modifying therapy emphasizes the need for cost-effective and scalable methods to identify individuals likely to have AD and benefit from AD drugs.

Online, self-administered, digital neuropsychological tests are one approach to identify individuals with cognitive impairments [9, 10]. Popular in-clinic tests such as the Clinical Dementia Rating, Montreal Cognitive Assessment, Cogstate Brief Battery, and Cambridge Neuropsychological Test Automated Battery (CANTAB^®^) have been developed as online versions [11–15]. These may be useful for early detection of cognitive decline in cognitively unimpaired (CU) adults [16], providing accessibility, efficiency, and diagnostic accuracy for dementia [9, 10, 17]. The CANTAB paired associates learning (PAL) neuropsychological test assesses visual learning and episodic memory, which are among the initial symptoms of AD [18]. Patients with mild cognitive impairment (MCI) and AD show poorer performance on PAL compared to CU adults [19–22]. PAL differentiates patients with MCI from age-matched CU individuals with a sensitivity of 0.83 and specificity of 0.82 [23]. PAL also has been shown to predict progression to dementia [19–21, 24] and correlates with AD biomarkers such as cerebrospinal fluid amyloid β and tau [25], hippocampal volume and activation [25, 26], and amyloid β positivity in PET scans [27]. The PAL online version showed comparable agreement with in clinic-based assessment [14]. A report from the Brain Health Registry (BHR), an online platform for aging and neuroscience research [28], showed that remotely collected PAL showed moderate feasibility and good construct validity [29].

Another approach is to identify individuals with subjective cognitive decline (SCD), such as self-reported memory problems in CU people [30]. The Everyday Cognition scale (ECog) is a well-established instrument which collects self-report and study partner-reported subjective information about cognitive change and instrumental activities of daily living which relate to cognitive function [31]. The ECog is especially effective in CU people [32, 33], and various cut-off points have been suggested for detecting MCI or AD in several older adult cohorts [33–35]. The BHR is utilizing ECog to detect SCD, with remotely collected ECog showing comparable scores to in-clinic assessments [36]. This measure is associated with cognitive function, probability of diagnosis of dementia, and AD biomarkers in cross-sectional studies [37, 38].

Subjective change measures are well known to be associated with cognitive decline and AD progression in in-clinic settings [39–42]. Remotely collected caregiver-reported SCD has been shown to be associated with future in-person assessed cognitive decline and dementia [43]. Despite the importance of subjective cognitive measures, however, there is relatively little information concerning the ability of subjective cognitive change to predict future cognitive decline in remote, online settings.

Therefore, the overall goal of this study was to investigate the association between SCD and longitudinal objective cognition in an unsupervised and online setting. Specifically, our aim was to investigate the association between subjective cognitive change and future cognitive decline in terms of both continuous change and determined dichotomized status. We hypothesized that ECog categorizations, previously established in in-clinic cohorts for detecting cognitive impairment, would predict changes in longitudinal PAL performance and identify older adults who were ‘cognitive decliners’, those who showed a significant declining slope (defined as lowest 5th percentile) in PAL scores, among our study participants in an online setting.

## Methods

### Participants

Participants were from the BHR database, which is an online neuroscience registry for developing cohorts focusing on cognitive aging. BHR participants provide electronic informed consent and complete self-report surveys and unsupervised online cognitive tests every six months [28, 44]. BHR launched in 2014 and the number of participants enrolled in BHR has reached 100,000 at March 2024. Additionally, caregivers or family members invited by participants can enroll separately into BHR as study partners and answer questionnaires about participants’ cognition every six months [45]. The BHR study is approved by the UCSF Institutional Review Board.

This study included participants meeting the following criteria: (i) completed PAL test one or more times; (ii) age at baseline PAL test ≥ 55; (iii) age at any longitudinal PAL tests ≤ 90; (iv) completed ECog questionnaire within six months of baseline PAL. According to the inclusion criteria, this study used data collected from March 2021, when PAL was newly implemented in BHR, to December 2023, the time of analysis.

### Sociodemographic and clinical measures

Baseline demographic information included age, gender (male, female, other, and prefer not to say), and years of education. In terms of ethnocultural background, we categorized participants into the following groups: non-Latinx African American, Latinx, non-Latinx White, and all others.

We used participants’ self-report of medical history including the diagnosis of MCI, dementia, and AD (“Please indicate whether you currently have or have had any of the following conditions in the past.”), history of taking medication for dementia (“Are you currently taking any of the following medications?”; donepezil, rivastigmine, galantamine, and memantine), and family history of AD (“Do you have any biological parents, full siblings, or biological children who have been diagnosed with Alzheimer’s Disease?”). We also included participant scores on an online adaptation of the Geriatric Depression Scale-Short Form (GDS) [46]. The GDS item regarding memory problems (“Do you feel you have more problems with memory than most?”) was excluded in this study as it might confound the results. The total score of GDS in this study ranged from 0 to 14 with higher score meaning more depressive symptoms. We also collected the status of subjective memory concern, focusing on the worries about subjective cognitive change, assessed with a yes or no question: “Are you concerned that you have a memory problem?”

### Everyday cognition scale

ECog scores at baseline were used in this study. ECog is a 39-item scale measuring subjective changes in instrumental activities of daily living compared to 10 years before [31]. Every item is rated on a 1–4 Likert scale (1 = no change or better; 2 = questionable or occasionally worse; 3 = consistently a little worse; 4 = consistently much worse) and the total score is the average of all responses ranging from 1 to 4 with a higher score meaning more decline. The original version of ECog, implemented in BHR, was analyzed in this study [31]. ECog ratings can be completed either by the individual themselves (self-reported ECog or Self-ECog) or by a study partner (study partner-reported ECog or SP-ECog). SP-ECog was used in this study when it was available.

In addition to the total ECog score, we also evaluated additional ECog scoring outcomes for predicting cognitive impairment [33–35]. We categorized ‘Self-ECog positive’ using cut-off score 1.31 or higher (impaired vs. normal) and ‘SP-ECog positive’ using cut-off score 1.36 or higher (impaired vs. normal) from studies by Rueda et al. [34]. and van Harten et al. [35]. We also categorized ‘ECog consistent’, defined as the respondent indicating any ECog item ≥ 3 (consistently worse) as shown in the study by van Harten et al. [35]. These categorizations have shown optimal performance in discriminating or predicting cognitive impairments in in-clinic cohorts.

### Paired associates learning

All participants are invited to complete the PAL online test every six months. Longitudinal PAL scores starting from the same baseline as ECog were used in this study. The PAL task is one of the CANTAB assessments developed by the University of Cambridge [47].

In the version of PAL used in the BHR, participants in the registry have the chance to undergo five stages of assessment, wherein they are tasked with learning two, four, six, eight, or twelve pattern-location pairings. Initially, participants are presented with boxes in a randomized sequence, each revealing either a pattern or an empty space, which they must commit to memory. Subsequently, patterns are displayed one at a time in the center of the screen, and participants must identify the box where each pattern was originally located. In the event of an error, the boxes reopen, allowing the participant up to four attempts to correctly recall the pattern locations. Progression to a more difficult stage is contingent upon correctly recalling all patterns in the current stage. Failure to complete a stage after four attempts results in the termination of the task.

PAL outcome measures were the number of correct answers at the first attempt (first attempt memory score, FAMS) and the number of errors made by the participants plus the estimated number of errors the participants would have made in any trials not reached due to early termination (total errors adjusted, TEA). These two measures are independent of each other and the most reported measures in previous literature [47]. Data cleaning steps excluded only participants who were unable to begin the PAL test due to challenges in understanding the online instructions or navigating the digital platform.

### Decliner

Additionally, we identified “decliner” groups based on separate evaluations of the PAL FAMS and PAL TEA scores. By examining individual slopes of PAL FAMS progression, participants with slopes equal to or lower than the fifth percentile, relative to the group’s average slope, were categorized as experiencing cognitive decline. Conversely, for PAL TEA, participants with slopes exceeding the 95th percentile, compared to the group’s slope, were deemed decliners. This analysis involved participants with at least two longitudinally assessed PAL scores available (*n* = 10,065). We followed a method used in previous studies for other computerized cognitive tests and unsupervised cognitive test batteries [48, 49], which involved inspecting slopes for longitudinal change to identify participants who were statistically abnormal, adjusting for the absence of any longitudinal change in performance or improvement as a learning effect [49, 50].

### Statistical analysis

For descriptive statistics, we employed independent t-tests for continuous variables and chi-squared tests for dichotomous variables. We also conducted linear regression analysis between clinical and demographic variables and PAL scores at baseline.

First, we aimed at determining the predictive capacity of baseline ECog outcomes—ECog positive, ECog consistent, and ECog total score—on PAL score longitudinal changes. We examined time interactions to assess the relationship between baseline ECog outcomes and longitudinal PAL progression in a linear mixed effect model. PAL scores served as the dependent variables, while ECog*time (month) and baseline PAL score were fixed effect variables in model 1. Additional variables including age at each PAL test, gender, years of education, race, GDS, family history of AD, AD medication use, and self-reported any impairment were included as fixed-effect variables in model 2. As we used several ECog outcomes as the dependent variables, false discovery rate corrections were conducted using Benjamini-Hochberg method [51]. We also calculated marginal R-squared (R²m) to evaluate the explanatory power of fixed effects in the linear mixed model [52]. The linear mixed models were also re-analyzed in participants excluding those who self-reported any impairment.

Second, we aimed at predictive capacity of baseline ECog outcomes on the ‘cognitive decliners’ status. We conducted multivariate logistic regression analyses to estimate associations between baseline ECog outcomes and the status of ‘decliners’, individuals exhibiting PAL progression slopes corresponding to the fifth percentile and below. For each ECog variable, separate multivariable logistic regression models to predict decliners were conducted, with age, gender, years of education, race, and the baseline PAL score included as additional predictors. We also calculated the odds ratios (OR) for predicting PAL decliners based on subjective memory concern, GDS, AD medication use, and self-reported any impairment. OR and 95% confidence intervals (CI) were calculated based on the logistic regression models. Additionally, the logistic regression models were re-analyzed in participants excluding those who self-reported any impairment. We used R version 4.3.2 (R Core Team, 2023) and SAS 9.4 (SAS Institute, Cary NC) in all analyses.

## Results

### Participants

**Fig. 1:**
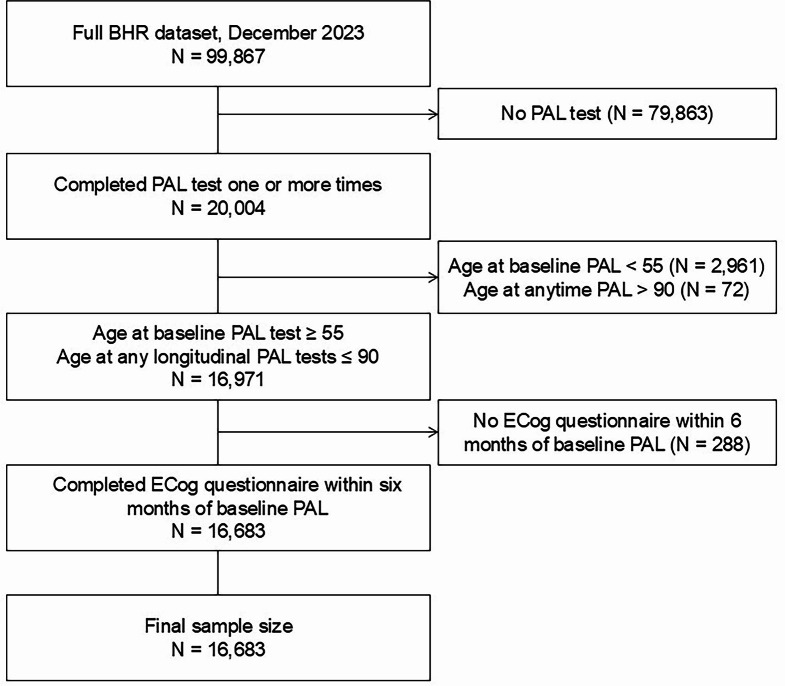
Flow chart for participant inclusion/exclusion. BHR, Brain Health Registry; PAL, paired associates learning; ECog, Everyday cognition scale

The total of 16,683 participants were included in this study. The flow chart illustrating the participant selection process based on the study’s inclusion and exclusion criteria is presented in Fig. 1. Baseline demographic and clinical characteristics of the participants are presented in Table 1. Among these participants, the mean age was 69.07 ± 7.34 years and the mean for years of education was 16.66 ± 2.26. The majority of the participants were non-Latinx white (88.8%) and female (72.0%). They were followed for an average of 2.52 ± 1.63 times (min 1, max 6) for 11.49 ± 11.53 (min 0, max 33) months, which provided 42,049 person-visits data in total.

**Table 1:** Baseline descriptive statistics

Variable	Total (*n* = 16,683)	Self-ECog positive (*n* = 7,378)	Self-ECog negative (*n* = 9,305)	t or x^2^, *p*
Age	69.07 ± 7.34 (55–90)	69.21 ± 7.69 (55–90)	68.53 ± 7.29 (55–90)	-5.85, < 0.001
Gender (female)	12,018 (72.0%)	5247 (71.1%)	6771 (72.8%)	5.48, 0.019
Years of education	16.66 ± 2.26 (6–20)	16.44 ± 2.32 (6–20)	16.64 ± 2.28 (6–20)	5.70, < 0.001
Race: Latinx	969 (5.8%)	488 (6.6%)	481 (5.2%)	15.44 < 0.001
Race: Non-Latinx African American	364 (2.2%)	159 (2.2%)	205 (2.2%)	0.02, 0.875
Race: Non-Latinx White	14,815 (88.8%)	6470 (87.7%)	8345 (89.7%)	16.19, < 0.001
Race: All other	535 (3.2%)	261 (3.5%)	274 (2.9%)	4.47, 0.035
Subjective memory concern	7263 (43.5%)	5062 (68.6%)	2201 (23.7%)	3381.4, < 0.001
GDS	2.37 ± 2.79 (0–14)	3.71 ± 3.37 (0–14)	1.70 ± 2.22 (0–14)	-42.68, < 0.001
Family history of AD	7289 (43.7%)	3288 (44.6%)	4001 (43.0%)	4.04, 0.044
AD medication use	219 (1.3%)	151 (2.1%)	68 (0.7%)	53.99, < 0.001
Self-reported MCI	1494 (9.0%)	1231 (16.7%)	263 (2.8%)	967.63, < 0.001
Self-reported AD	206 (1.2%)	155 (2.1%)	51 (0.6%)	80.09, < 0.001
Self-reported dementia	272 (1.6%)	209 (2.7%)	63 (0.7%)	100.84, < 0.001
Self-reported any impairment	1550 (9.3%)	1270 (17.2%)	280 (3.0%)	983.47, < 0.001
Self-ECog	1.39 ± 0.42 (1–4)	1.76 ± 0.46 (1.31–4)	1.14 ± 0.10 (1–1.31)	-114.22, < 0.001
SP-ECog	1.27 ± 0.37 (1–4)	1.44 ± 0.50 (1–3.75)	1.20 ± 0.29 (1–4)	-16.22, < 0.001
PAL FAMS	11.71 ± 4.22 (0–20)	11.29 ± 4.33 (0–20)	12.05 ± 4.11 (0–20)	11.44, < 0.001
PAL TEA	44.83 ± 29.43 (0–113)	47.81 ± 29.86 (0–113)	42.47 ± 28.86 (0–113)	-11.65, < 0.001

Data are mean ± standard deviation (min-max) or *n* (%)

Self-reported any impairment incorporates any report of MCI, AD, and dementia

ECog, Everyday cognition scale; GDS, geriatric depression scale; MCI, mild cognitive impairment; AD, Alzheimer’s disease; SP, study partner; PAL, paired associates learning; FAMS, first attempt memory score; TEA, total errors adjusted

### Comparisons of clinical variables between ECog positive and negative groups

Participants were divided into Self-ECog positive (*n* = 7,378) and negative (*n* = 9,305) using the cut-point of Self-ECog total score 1.31 [34]. Compared to the Self-ECog negative group, the Self-ECog positive group had a higher percentage of individuals with subjective memory concerns, higher GDS scores (indicating more depressive symptoms), and higher percentages of a self-reported diagnosis of cognitive impairment (MCI, AD, and dementia), and AD medication use (Table 1). Self-ECog scores differed significantly between the two groups (1.76 ± 0.46 in the positive and 1.14 ± 0.10 in the negative group, *t* = -114.22, *p* < 0.001) and SP-ECog scores also differed (1.44 ± 0.50 in the positive and 1.20 ± 0.29 in the negative group, *t* = -16.22, *p* < 0.001). Baseline PAL scores were worse in Self-ECog positive group with lower score in PAL FAMS (*t* = 11.44, *p* < 0.001) and higher score in PAL TEA (*t* = -11.65, *p* < 0.001) (Table 1).

### Baseline association between demographic and clinical variables and PAL scores

Demographic and clinical variables associated with PAL FAMS and TEA are presented in Table S1 in supplemental data. Demographic factors such as younger age (estimate = -0.164, *p* < 0.001), female gender (estimate = 0.771, *p* < 0.001), higher education (estimate = 0.098, *p* < 0.001), and non-Latinx white race were associated with better PAL FAMS. Clinical factors such as having subjective memory concern (estimate = -0.373, *p* < 0.001), higher GDS (estimate = -0.052, *p* < 0.001), AD medication use (estimate = -0.690, *p* = 0.014), and having self-reported cognitive impairment (estimate = -1.458, *p* < 0.001) were associated with worse PAL FAMS. However, Self-ECog positive was not associated with either baseline PAL score (FAMS: estimate = -0.067, *p* = 0.370; TEA: estimate = 0.533, *p* = 0.306). Standardized estimates were also calculated and presented in Table S1 in supplemental data.

### Longitudinal PAL scores

**Fig. 2:**
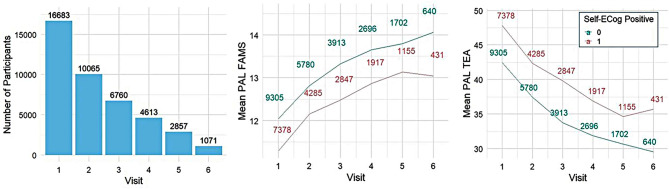
Participants’ observed follow-ups and mean PAL scores at each visit. PAL, paired associates learning; FAMS, first attempt memory score; TEA, total errors adjusted; ECog, Everyday cognition scale

The number of participants at each visit and the progression of mean PAL scores, stratified by Self-ECog positive status, are presented in Fig. 2.  

### Effect of baseline ECog outcomes on the longitudinal PAL scores

**Fig. 3:**
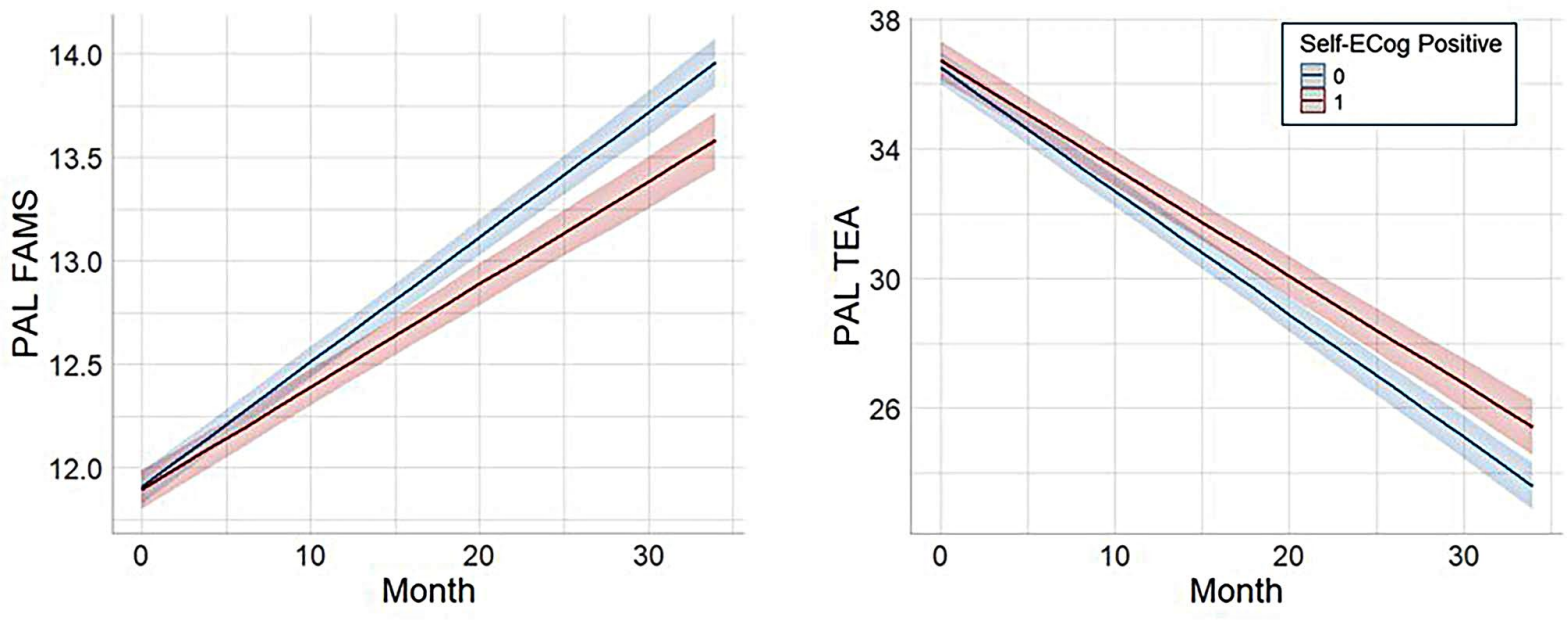
Predicted trajectory of PAL scores in groups stratified by Self-ECog positive. Trajectory of PAL scores in groups stratified by Self-ECog positive (total score ≥ 1.31) status: The regression lines and the 95% confidence intervals for the predicted scores in linear mixed model adjusted for age, gender, years of education, race, GDS, family history of AD, AD medication use, self-reported any impairment, and baseline PAL score. PAL, paired associates learning; FAMS, first attempt memory score; TEA, total errors adjusted; ECog, Everyday cognition scale; GDS, geriatric depression scale; AD, Alzheimer’s disease

The results of linear mixed effect model with time interactions of the baseline ECog outcomes on the longitudinal PAL scores are presented in Table 2. Self-ECog positive and Self-ECog consistent showed significant associations with decline in PAL FAMS in model 1 adjusted for the baseline PAL score (Self-ECog positive: estimate = -0.010, *p* < 0.001; Self-ECog consistent: estimate = -0.008, *p* = 0.004) and model 2 adjusted for age, gender, years of education, race, GDS, family history of AD, AD medication use, self-reported any impairment, and baseline PAL score (Self-ECog positive: estimate = -0.011, *p* < 0.001; Self-ECog consistent: estimate = -0.009, *p* = 0.002). Self-ECog positive also predicted longitudinal change of PAL TEA score in both models (Model 1: estimate = 0.046, *p* = 0.005; Model 2: estimate = 0.046, *p* = 0.006). They all showed good model fit with large explanatory power of fixed effects (R²m 0.559–0.661). Predicted trajectories of both PAL scores by Self-ECog positive in model 2 are presented in Fig. 3. Neither Self-ECog total score nor any SP-ECog total scores were associated with longitudinal change in PAL scores after adjusting multiple comparisons. However, when the linear mixed model was applied to participants without self-reported any impairment, both the Self-ECog total and SP-ECog total scores showed significant time interactions with longitudinal PAL FAMS and PAL TEA scores, whereas the dichotomized ECog scores showed weaker effects (Table S2 and Figure S1).

**Table 2:** Time interactions of ECog scores on longitudinal PAL scores in linear mixed effect model (*n* = 16,683)

Variable	PAL FAMS			PAL TEA score		
	Estimate (SE)	*p* value	*R*²m	Estimate (SE)	*p* value	*R*²m
Model 1						
Self-ECog positive*Time (month)	-0.010 (0.003)	< 0.001*	0.562	0.046 (0.017)	0.005*	0.661
Self-ECog consistent*Time	-0.008 (0.003)	0.004*	0.562	0.038 (0.016)	0.020	0.660
Self-ECog total*Time	-0.006 (0.003)	0.062	0.562	0.026 (0.020)	0.185	0.661
SP-ECog positive*Time	-0.001 (0.007)	0.842	0.526	0.018 (0.041)	0.658	0.640
SP-ECog consistent*Time	-0.004 (0.007)	0.581	0.526	0.019 (0.039)	0.627	0.640
SP-ECog total*Time	-0.013 (0.008)	0.096	0.528	0.097 (0.048)	0.042	0.642
Model 2						
Self-ECog positive*Time	-0.011 (0.003)	< 0.001*	0.559	0.046 (0.017)	0.006*	0.658
Self-ECog consistent*Time	-0.009 (0.003)	0.002*	0.559	0.039 (0.017)	0.020	0.658
Self-ECog total*Time	-0.006 (0.003)	0.073	0.559	0.024 (0.020)	0.235	0.658
SP-ECog positive*Time	-0.003 (0.007)	0.620	0.525	0.031 (0.042)	0.465	0.639
SP-ECog consistent*Time	-0.004 (0.007)	0.500	0.525	0.024 (0.040)	0.537	0.639
SP-ECog total*Time	-0.015 (0.008)	0.061	0.526	0.105 (0.049)	0.030	0.640

Each variable was separately put in a multivariable linear mixed effect model

Model 1: adjusted for baseline PAL score

Model 2: adjusted for age, gender, years of education, race, geriatric depression scale, family history of AD, AD medication use, self-reported any impairment, and baseline PAL score

*p values that survived false discovery rate correction using Benjamini-Hochberg method

ECog, Everyday cognition scale; PAL, paired associates learning; FAMS, first attempt memory score; TEA, total errors adjusted; SE, standard error; R²m, marginal R-squared; Self-ECog, self-reported ECog; SP-ECog, study partner-reported ECog; AD, Alzheimer’s disease

### Effect of baseline ECog outcomes on PAL decliner status

**Fig. 4:**
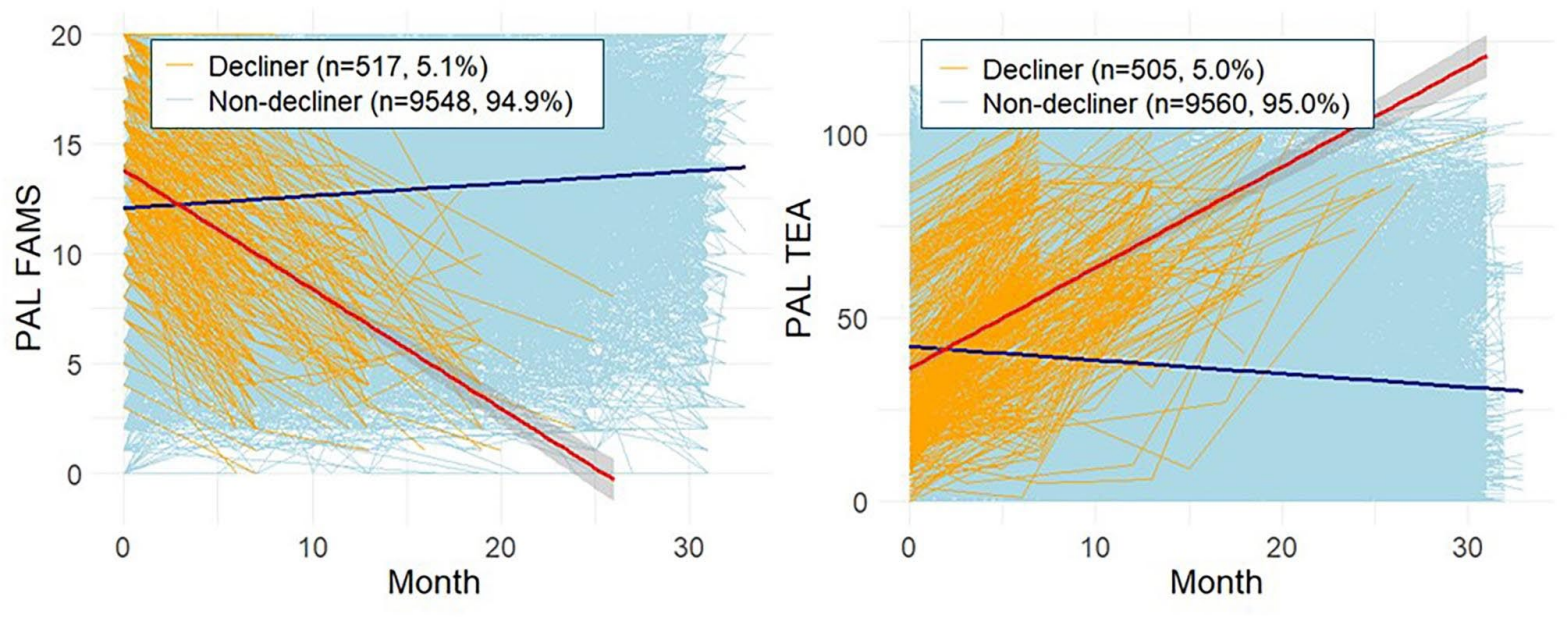
Predicted trajectory of PAL scores in decliner and non-decliner groups. Trajectory of PAL scores in decliner and non-decliner groups: Linear regression lines in each group are overlaid on individual spaghetti plots in groups stratified by decliner status. Decliners were identified based on PAL FAMS slopes over time at or below the fifth percentile relative to the group mean slope, while PAL TEA decliner was defined as 95th percentile or higher. The analysis was conducted using only participants who had at least two PAL scores available (*n* = 10,065). PAL, paired associates learning; FAMS, first attempt memory score; TEA, total errors adjusted; ECog, Everyday cognition scale; GDS, geriatric depression scale; AD, Alzheimer’s disease

Among the 10,065 participants who completed at least two PAL tests, 517 participants (5.1%) were identified as PAL decliners, showing the worst slope of PAL progression corresponding to the fifth percentile and below, based on PAL FAMS scores, and 505 participants (5.0%) were identified based on PAL TEA scores. The spaghetti plots of the decliners and non-decliners with groupwise regression lines and 95% CIs are presented in Fig. 4.

ORs for identifying PAL decliner by ECog scores using logistic regression models are presented in Table 3. For PAL FAMS decliners, most Self- and SP-ECog outcomes showed significant associations with ORs ranging 1.173–2.417. For identifying PAL TEA decliners, three Self-ECog outcomes showed significant associations (OR 1.200–1.466). Additionally, baseline clinical variables including subjective memory concern, higher GDS scores, and self-reported any impairment were associated with higher probability of being a PAL decliner. In the analysis of participants without self-reported any impairment, SP-ECog variables showed the same significant results, whereas Self-ECog Positive and Self-ECog Consistent did not show significant associations with Decliner status on PAL FAMS (Table S3).

**Table 3:** Odds ratios for predicting PAL decliner by ECog and other variables using logistic regression model (*n* = 10,065)

Variable	Odds ratio (95% CI)	
	Decliner on PAL FAMS (*n* = 517)	Decliner on PAL TEA (*n* = 505)
Self-ECog positive (≥ cut-off 1.31)	1.173 (0.977–1.407)	1.200 (1.000-1.439)*
Self-ECog consistent (any item ≥ 3)	1.262 (1.053–1.513)*	1.320 (1.102–1.583)*
Self-ECog total	1.390 (1.121–1.708)*	1.466 (1.194–1.783)*
SP-ECog positive (≥ cut-off 1.36)	2.128 (1.368–3.267)*	0.692 (0.385–1.172)
SP-ECog consistent	2.417 (1.591–3.655)*	1.195 (0.749–1.862)
SP-ECog total	1.832 (1.071–2.964)*	0.704 (0.326–1.346)
Subjective memory concern	1.597 (1.328–1.920)*	1.511 (1.256–1.818)*
GDS	1.062 (1.029–1.095)*	1.061 (1.029–1.094)*
AD medication use	0.984 (0.379–2.093)	0.763 (0.268–1.703)
Self-reported any impairment	1.502 (1.091–2.027)*	1.618 (1.196–2.150)*

For each variable, separate multivariable logistic regression models for predicting decliners were conducted, with age, gender, years of education, race, and the baseline PAL score included as additional predictors

The reference groups were the Non-decliner groups

Decliners were identified based on PAL FAMS slopes at or below the fifth percentile relative to the group mean slope, while PAL TEA decliner was defined as 95th percentile or higher

*significant

PAL, paired associates learning; ECog, Everyday cognition scale; CI, confidence interval; FAMS, first attempt memory score; TEA, total errors adjusted; Self-ECog, self-reported ECog; SP-ECog, study partner-reported ECog; GDS, geriatric depression scale; AD, Alzheimer’s disease

## Discussion

The major findings of this study of older adults enrolled in the BHR were: First, subjective cognitive change positive status, defined using a previously established ECog cut-off or categorization for identifying those with cognitive impairment, was associated with longitudinal decline in performance on the PAL visual learning and memory test. Second, greater subjective decline, defined using both continuous and dichotomous ECog scoring outcomes, was associated with higher probability of being a PAL decliner (those with PAL progression slopes in the fifth percentile). These results support our hypothesis that online, self-administered measures of subjective cognitive change have the potential to identify older adults who are likely to have objective cognitive decline. Our findings demonstrate that this online assessment approach might be useful to identify older adults with or at risk for cognitive impairment and clinical progression.

The first major finding of this study is that greater self-report of subjective cognitive and functional decline, measured using ECog, was associated with greater future longitudinal decline in visual learning and memory, assessed using the PAL test. Specifically, Self-ECog positive and Self-ECog consistent were associated with greater decline in PAL. The continuous ECog total score, on the other hand, showed a stronger association with PAL progression than the dichotomized ECog in participants without self-reported any impairment. These results may highlight the utility of ECog dichotomization, which was previously established as an effective method for discriminating individuals with MCI or dementia from CU individuals. The fact that both dichotomized and continuous ECog scores from in-clinic cohorts were associated with PAL progression in an online registry suggests that a simple subjective cognitive change measure could be useful for predicting future objective cognitive decline in an online, unsupervised setting. This finding is different from the results of previous studies that investigated the predictive ability of ECog for diagnosis of MCI or AD [34, 35, 53], that investigated the association between the in-clinic and pen-and-paper ECog score and clinical diagnosis. Although online registries are efficient means to collect repeated measures pertaining to cognitive function, there have been few studies of longitudinal cognitive assessment in online setting. Banh et al. previously reported longitudinal Cogstate brief battery in association with self-reported MCI and subjective memory concern in BHR [48], and Stricker et al. reported longitudinal at-home Cogstate brief battery results in comparison with in-clinic results [54]. We analyzed longitudinal online PAL scores over an average duration of 11.49 months and found significant monthly changes in PAL scores in relation to ECog dichotomizations. While the large sample size may have contributed to the significance of the results, the initial differentiation with a good model fit suggests that these findings could become more pronounced in future longitudinal studies with longer follow-up periods.

However, SP-ECog scores were less associated with longitudinal PAL scores than Self-ECog scores in this study, which contrasts with previous studies that demonstrated better predictive power of SP-ECog for diagnosing MCI or AD compared to Self-ECog [34, 35]. Two aspects of our study design likely contribute to this finding. First, BHR has a selection bias for older adults who are CU or who have mild impairment with intact functional abilities. We believe that this is because more impaired people are less likely to have the ability and motivation to join BHR themselves, compared to CU people. In fact, the demographics of our sample, which is overwhelmingly composed of CU people, support this bias. In this unimpaired or mildly impaired group, it is likely that changes in cognition and function would be subtle, and not observable by study partners. Second, BHR does not require a minimum amount of contact of familiarity between participants and study partners for the study partner to qualify for the study. Therefore, the lack of signal for SP-ECog may be, in part, driven by lower levels of familiarity between participant and study partner than in other studies. Still, previous BHR studies have shown that SP-ECog is cross-sectionally associated with objective learning and memory as well as with self-report of a diagnosed cognitive impairment [45]. Therefore, the role of SP-ECog in identifying cognitive decline warrants further study in this sample.

The second major finding of this study is that greater subjective cognitive change was associated with greater odds of being a PAL ‘decliner’, defined as those who exhibited the worst PAL progression slopes corresponding to the fifth percentile and below. This is partly in line with the previous studies that showed significant association between ECog and diagnosis of MCI or AD. Although decliner status is not a diagnosis of cognitive impairment, definition by lowest fifth percentile was often used as an impaired status in online cognitive tests [48, 49] as well as traditional neuropsychological tests [55, 56]. Thus, this result supports the hypothesis that ECog may help identify those with cognitive impairment. However, there were differences between Self-ECog and SP-ECog in association with decliner status. Self-ECog outcomes demonstrated significant ORs for decliners based on both FAMS (memory scores) and TEA (error scores), whereas SP-ECog categorizations only showed significant OR for decliners based on FAMS, with higher OR values compared to Self-ECog outcomes. We believe this may be because individuals who exhibit decline based on memory score progressions are more likely to have impairments such as MCI or AD. These individuals might be more easily observed by study partners or may have impaired awareness of their illnesses [57].

This study also found that the ECog predicted cognitive decliner status as effectively as other clinical variables. Subjective memory concern, assessed with a simple yes/no question about worries regarding cognitive change, showed a strong association with decliner status, supporting previous findings that anxiety about SCD may be linked to future cognitive impairments [39, 58]. Similarly, self-reported MCI or dementia increased the odds of being classified as a cognitive decliner, highlighting the reliability of online-collected diagnostic information in predicting future cognitive decline [48]. Depressive symptoms, measured by the GDS, were also associated with cognitive decliner status, with a small but significant effect. This is consistent with the understanding that depressive symptoms can both be a risk factor and an early sign of neurodegenerative conditions like Alzheimer’s disease [59]. This study demonstrated that, similar to other clinical variables, the ECog scale was a strong predictor of cognitive decliner status. Specifically, subjective memory concern, self-reported MCI or dementia, and depressive symptoms all exhibited significant associations with decliner status, highlighting the ECog’s comparable predictive power in relation to established clinical measures.

An examination of ECog and PAL performance in our study supports the validity of the remote, online assessment approach, and also highlights important selection biases. Compared to other clinical samples used to study ECog, our participants are younger, have higher education levels, a higher proportion are female, and lacks sufficient ethnocultural diversity [34, 35, 53]. Additionally, our sample lacks traditional baseline cognitive assessments. However, the mean Self-ECog score in CU group in this study (1.36 ± 0.37) was comparable with CU individuals in previous in-clinic cohorts (1.37 ± 0.46 [33]; 1.34 ± 0.31 [34]; 1.2 (1.1–1.5) [35]; 1.46 ± 0.47 [53]). Self-ECog positivity also showed association with various clinical information such as subjective memory concern, depressive symptoms, and self-reported MCI, AD and dementia, and taking medications for AD in this study, which corresponds with previous in-clinic studies [34, 35, 53, 60]. These results further support the validity of unsupervised online ECog, as first addressed in a previous head-to-head comparison with in-clinic version [36].

In our study, longitudinal PAL tests showed an improvement in scores over time. The practice effect is a common phenomenon in repeated neuropsychological tests [50] and this was also found in online longitudinal cognitive assessments studies [48, 54]. The practice effect was prominent in the first two sessions [48] and lasted for three to four sessions [50] in previous studies. PAL test also showed practice effect even in cognitively impaired participants [61]. Our results are in line with these prior studies showing most improvement between first two sessions and begin to deteriorate from session six in PAL FAMS in Self-ECog positive group. Although caution is needed when interpreting the current online PAL scores in our sample, this study provides evidence of useful online measurement of cognitive change, in a large sample size (*n* = 16,638).

This study also has several limitations, with the most significant being selection biases resulting from the recruitment methods used in BHR. As a fully online study, participants are more likely to have higher internet literacy and heightened concerns about their cognitive health, which may not represent the broader population. Additionally, there is a skewed distribution in demographic factors, particularly race, ethnicity, and socioeconomic status, further limiting the generalizability of the findings. We have previously observed that certain groups, such as individuals from non-white racial backgrounds, Latino communities, and those with lower educational attainment, are more likely to drop out of follow-up assessments [62]. These patterns introduce additional bias and reduce the representativeness of the data. A second limitation is that the large sample size likely contributed to statistically significant associations. The third limitation is that this study relies on self-reported information and lacks clinical diagnosis or AD biomarker data, which prevents us from distinguishing between individuals with AD dementia, MCI due to AD, and other forms of cognitive impairment. A fourth limitation is that the PAL FAMS and PAL TEA scores were analyzed using raw scores without excluding data from participants with extremely poor performance. However, participants who were unable to start the PAL test due to digital literacy issues were excluded. Additional analyses, which were limited to individuals without self-reported any impairment, yielded similar results, supporting the robustness of our findings.

## Conclusions

In the BHR’s unsupervised online setting, ECog demonstrated feasibility in predicting longitudinal decline in PAL scores, both in terms of continuous changes and when dichotomized as decliners versus non-decliners. These findings underscore the value of subjective cognitive change measures to identify those at risk for cognitive decline. Also, this study shows the utility of previously dichotomized ECog cut-off or categorizations in an online setting. Taken together, unsupervised, remotely collected SCD might have the potential to identify individuals with cognitive decline, who are likely to be at risk for or in the early stages of cognitive impairment.

## Electronic supplementary material

Below is the link to the electronic supplementary material.

Supplementary Material 1

## Data Availability

The datasets used and/or analysed during the current study are available from the corresponding author on reasonable request.

## Abbreviations

ECog: Everyday Cognition Scale
BHR: Brain Health Registry
CU: Cognitively Unimpaired
PAL: Paired Associates Learning
Self-ECog: Self-reported ECog
SP-ECog: Study Partner-reported ECog
FAMS: First Attempt Memory Score
TEA: Total Errors Adjusted
AD: Alzheimer’s Disease
PET: Positron Emission Tomography
CANTAB: Cambridge Neuropsychological Test Automated Battery
SCD: Subjective Cognitive Decline
MCI: Mild Cognitive Impairment
GDS: Geriatric Depression Scale-Short Form
R²m: R-squared
OR: Odds Ratios
CI: Confidence Intervals
