# Supplementary material for: Subjective cognitive decline predicts longitudinal neuropsychological test performance in an unsupervised online setting in the Brain Health Registry
Source: Alzheimers Res Ther. 2025 Jan 7;17:10. doi: 10.1186/s13195-024-01641-2 (PMC11706033; doi:10.1186/s13195-024-01641-2)
Supplement: Supplementary file 1 — Supplementary Material 1 [file 13195_2024_1641_MOESM1_ESM.docx]

| **Table S1**. Association between demographic and clinical information and PAL scores at baseline (*n* = 16,683) | | | | | | | | |
| --- | --- | --- | --- | --- | --- | --- | --- | --- |
| **Variable** | **PAL FAMS** | | | | **PAL TEA score** | | | |
|  | **Estimate (SE)** | **Standardized Estimate (SE)** | ***p* value** | **Cohen’s *d*** | **Estimate (SE)** | **Standardized Estimate (SE)** | ***p* value** | **Cohen’s *d*** |
| Age | -0.164 (0.004) | -1.227 (0.033) | <0.001 | -37.198 | 1.238 (0.031) | 9.250 (0.031) | <0.001 | 40.533 |
| Gender (female) | 0.771 (0.073) | 0.771 (0.073) | <0.001 | 10.638 | -6.553 (0.502) | -6.553 (0.502) | <0.001 | -13.066 |
| Years of education | 0.098 (0.014) | 0.226 (0.033) | <0.001 | 6.936 | -0.790 (0.098) | -1.813 (0.225) | <0.001 | -8.052 |
| Race: Latin (ref: white) | -1.121 (0.140) | -1.121 (0.140) | <0.001 | -8.006 | 7.200 (0.969) | 7.199 (0.969) | <0.001 | 7.430 |
| Race: African American (ref: white) | -1.651 (0.221) | -1.651 (0.221) | <0.001 | -7.470 | 10.244 (1.529) | 10.244 (1.529) | <0.001 | 6.700 |
| Race: All other (ref: white) | -0.448 (0.184) | -0.448 (0.184) | 0.015 | -2.433 | 2.453 (1.273) | 2.454 (1.273) | 0.054 | 1.927 |
| Subjective memory concern | -0.373 (0.075) | -0.373 (0.075) | <0.001 | -4.972 | 3.015 (0.520) | 3.015 (0.520) | <0.001 | 5.802 |
| GDS | -0.052 (0.012) | -0.156 (0.035) | <0.001 | -4.469 | 0.370 (0.082) | 1.094 (0.242) | <0.001 | 4.525 |
| Family history of AD | -0.004 (0.065) | -0.002 (0.032) | 0.947 | -0.067 | 0.654 (0.452) | 0.325 (0.224) | 0.147 | 1.449 |
| Taking AD medication | -0.690 (0.280) | -0.079 (0.032) | 0.014 | -2.468 | 3.681 (1.934) | 0.419 (0.220) | 0.057 | 1.903 |
| Self-reported any impairment | -1.458 (0.117) | -1.458 (0.117) | <0.001 | -12.506 | 9.524 (0.807) | 9.524 (0.807) | <0.001 | 11.808 |
| Self-ECog positive | -0.067 (0.075) | -0.033 (0.037) | 0.370 | -0.897 | 0.533 (0.520) | 0.265 (0.258) | 0.306 | 1.024 |
| All variables were put together in the multivariable linear regression model (*F* = 198.1, *p* < 0.0001, *R^2^* = 0.1284 for PAL FAM; *F* = 215.7, *p* < 0.0001, *R^2^* = 0.1453 for PAL TEA). Estimate is a predictive value adjusted for other variables while Cohen’s d is an effect size unadjusted for other variables.  PAL, paired associates learning; FAMS, first attempt memory score; TEA, total errors adjusted; SE, standard error; GDS, geriatric depression scale; AD, Alzheimer’s disease; ECog, Everyday cognition scale | | | | | | | | |

| **Table S2**. Time interactions of ECog scores on longitudinal PAL scores in linear mixed effect model in CU participants (*n* = 15,133) | | | | | | | | |
| --- | --- | --- | --- | --- | --- | --- | --- | --- |
| **Variable** | **PAL FAMS** | | | | **PAL TEA score** | | | |
|  | **Estimate (SE)** | ***p* value** | **Cohen’s *d*** | **R²m** | **Estimate (SE)** | ***p* value** | **Cohen’s *d*** | **R²m** |
| Model 1 |  |  |  |  |  |  |  |  |
| Self-ECog positive*Time (month) | -0.009 (0.003) | 0.002* | -3.059 | 0.548 | 0.044 (0.018) | 0.012* | 2.506 | 0.650 |
| Self-ECog consistent*Time | -0.006 (0.003) | 0.048 | -1.978 | 0.548 | 0.035 (0.017) | 0.045 | 2.006 | 0.649 |
| Self-ECog total*Time | -0.011 (0.004) | 0.008* | -2.640 | 0.548 | 0.057 (0.024) | 0.018* | 2.371 | 0.650 |
| SP-ECog positive*Time | -0.009 (0.008) | 0.240 | -1.175 | 0.500 | 0.044 (0.045) | 0.332 | 0.970 | 0.623 |
| SP-ECog consistent*Time | -0.010 (0.007) | 0.148 | -1.447 | 0.500 | 0.039 (0.042) | 0.349 | 0.936 | 0.623 |
| SP-ECog total*Time | -0.033 (0.010) | 0.001* | -3.238 | 0.502 | 0.166 (0.061) | 0.006* | 2.741 | 0.624 |
| Model 2 |  |  |  |  |  |  |  |  |
| Self-ECog positive*Time | -0.009 (0.003) | 0.002* | -3.060 | 0.544 | 0.043 (0.018) | 0.016* | 2.406 | 0.646 |
| Self-ECog consistent*Time | -0.006 (0.003) | 0.039 | -2.060 | 0.544 | 0.034 (0.018) | 0.052 | 1.941 | 0.646 |
| Self-ECog total*Time | -0.010 (0.004) | 0.011* | -2.536 | 0.544 | 0.054 (0.025) | 0.027 | 2.211 | 0.646 |
| SP-ECog positive*Time | -0.010 (0.008) | 0.183 | -1.333 | 0.497 | 0.053 (0.046) | 0.246 | 1.160 | 0.622 |
| SP-ECog consistent*Time | -0.011 (0.007) | 0.137 | -1.487 | 0.498 | 0.042 (0.043) | 0.327 | 0.980 | 0.622 |
| SP-ECog total*Time | -0.035 (0.010) | <0.001* | -3.366 | 0.499 | 0.176 (0.062) | 0.004* | 2.861 | 0.622 |
| Each variable was separately put in a multivariable linear mixed effect model.  Model 1: adjusted for baseline PAL score  Model 2: adjusted for age, gender, years of education, race, geriatric depression scale, family history of Alzheimer’s disease, taking Alzheimer’s disease medication, self-reported any impairment, and baseline PAL score  **p* values that survived false discovery rate correction using Benjamini-Hochberg method  ECog, Everyday cognition scale; PAL, paired associates learning; CU, cognitively unimpaired; FAMS, first attempt memory score; TEA, total errors adjusted; SE, standard error; R²m, marginal R-squared; Self-ECog, self-reported ECog; SP-ECog, study partner-reported ECog | | | | | | | | |


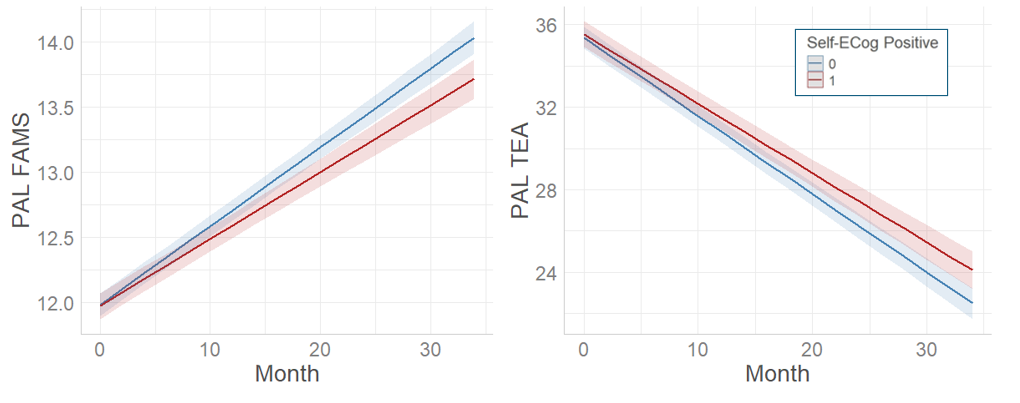
**Figure S1.** Predicted trajectory of PAL scores in groups stratified by Self-ECog positive in CU participants (*n* = 15,133)

Trajectory of PAL scores in groups stratified by Self-ECog positive (total score ≥ 1.31) status in CU participants: The regression lines and the 95% confidence intervals for the predicted scores in linear mixed model adjusted for age, gender, years of education, race, GDS, family history of AD, taking AD medication, self-reported any impairment, and baseline PAL score.

PAL, paired associates learning; FAMS, first attempt memory score; TEA, total errors adjusted; ECog, Everyday cognition scale; CU, cognitively unimpaired; GDS, geriatric depression scale; AD, Alzheimer’s disease

| **Table S3**. Odds ratios for predicting PAL decliner by ECog and other variables using logistic regression in CU participants (*n* = 9,205) | | |
| --- | --- | --- |
|  | **Odds ratio (95% confidence interval)** | |
| **Variable** | **Decliner on PAL FAMS** | **Decliner on PAL TEA** |
| Self-ECog positive (≥ cut-off 1.31) | 1.120 (0.922-1.359) | 1.139 (0.939-1.379)* |
| Self-ECog consistent (any item ≥ 3) | 1.182 (0.975-1.430) | 1.247 (1.031-1.508)* |
| Self-ECog total | 1.426 (1.099-1.828)* | 1.336 (1.032-1.708)* |
| SP-ECog positive (≥ cut-off 1.36) | 2.145 (1.349-3.356)* | 0.804 (0.446-1.364) |
| SP-ECog consistent | 2.501 (1.619-3.840)* | 1.231 (0.762-1.938) |
| SP-ECog total | 2.265 (1.230-3.936)* | 0.792 (0.347-1.597) |
| Subjective memory concern | 1.596 (1.313-1.938)* | 1.427 (1.175-1.732)* |
| GDS | 1.062 (1.026-1.097)* | 1.055 (1.020-1.090)* |
| Taking AD medication | 0.356 (0.020-1.654) | 0.309 (0.017-1.419) |
| For each variable, separate multivariable logistic regression models for predicting decliners were conducted, with age, gender, years of education, race, and baseline PAL score included as additional predictors.  The reference groups were the Non-decliner groups.  Decliners were identified based on PAL FAMS slopes at or below the fifth percentile relative to the group mean slope, while PAL TEA decliner was defined as 95th percentile or higher.  *significant  PAL, paired associates learning; ECog, Everyday cognition scale; CU, cognitively unimpaired; FAMS, first attempt memory score; TEA, total errors adjusted; SD, standard deviation; GDS, geriatric depression scale; AD, Alzheimer’s disease | | |

**Figure S2.** Predicted trajectory of PAL scores in decliner and non-decliner groups in CU participants (n = 9,205)


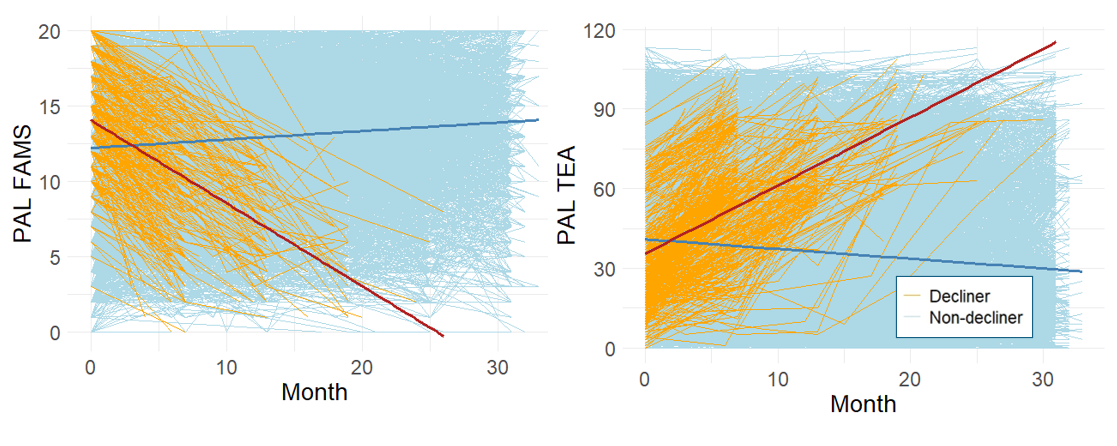


Trajectory of PAL scores in decliner and non-decliner groups in CU participants: Linear regression lines in each group are overlaid on individual spaghetti plots in groups stratified by decliner status. Decliners were identified based on PAL FAMS slopes over time at or below the fifth percentile relative to the group mean slope, while PAL TEA decliner was defined as 95th percentile or higher. The analysis was conducted using only CU participants who had at least two PAL scores available (*n* = 9,205).

PAL, paired associates learning; CU, cognitively unimpaired; FAMS, first attempt memory score; TEA, total errors adjusted; ECog, Everyday cognition scale; GDS, geriatric depression scale; AD, Alzheimer’s disease; CU, cognitively unimpaired
